# Copper Substitution and Noise Reduction in Brake Pads: Graphite Type Selection

**DOI:** 10.3390/ma5112258

**Published:** 2012-11-09

**Authors:** Raffaele Gilardi, Luigi Alzati, Mamadou Thiam, Jean-François Brunel, Yannick Desplanques, Philippe Dufrénoy, Sanjeev Sharma, Jayashree Bijwe

**Affiliations:** 1 Timcal Ltd., Strada Industriale 12, Bodio 6743, Switzerland; E-Mail: l.alzati@ch.timcal.com; 2 Laboratoire de Mécanique de Lille, Boulevard Paul Langevin, Villeneuve d’Ascq Cedex 59655, France; E-Mails: masseck.thiam@gmail.com (M.T.); jean-francois.brunel@polytech-lille.fr (J.-F.B.); yannick.desplanques@ec-lille.fr (Y.D.); philippe.dufrenoy@polytech-lille.fr (P.D.); 3 Indian Institute of Technology, New Delhi 110016, India; E-Mails: sonu82sharma@gmail.com (S.S.); jbijwe@itmmec.iitd.ac.in (J.B.)

**Keywords:** graphite, brake pad, noise, thermal conductivity

## Abstract

Graphite is commonly used in brake pads. The use of graphite powder has the main goal of solid state lubrication and friction coefficient stabilization. In this article results on resin bonded brake pads with focus on noise performance and heat dissipation are presented. Experimental tests are based on model friction materials with a known formulation and a reduced number of components for a better identification of the role of the graphite type. Results clearly indicate that both noise performance and thermal conductivity are strongly affected by the type of graphite. Guidelines for the selection of graphite types for optimized friction materials are given.

## 1. Introduction

A major focus of research in the automotive industry is passenger’s comfort during braking. Noisy brakes are still the first problem reported by consumers and it has a big impact on the perceived quality of the vehicle. Particularly unpleasant noise levels are at frequencies higher than 1000 Hz (squealing). Squeals are induced by the friction due to contact between the brake pads and disc. Squeal during braking is a complex issue that covers a wide field of dynamics at macroscopic scale (different components of the brake caliper) but also on a microscopic level (chemical composition of brake pads). While the coupling of vibrational modes leading to squeal are now well understood and identified, the conditions that cause squeal occurrences remain poorly understood. This is mainly due to the complexity of the physical phenomena underlying the contact between the surfaces and the many factors that influence local friction. In order to improve brake NVH (Noise, Vibration and Harshness) performance, it is essential to understand which elements cause the squealing.

Another focus of research in brake pads is generated by the new environmental regulations in the USA, aiming to reduce the amount of copper in brake pads initially to less that 5% and eventually to less than 0.5%. Copper has unique properties and functionalities in brake pads (friction stability, wear resistance, heat dissipation, noise damping). Among the 20 to 30 different ingredients contained in a typical brake pad formulation, there is no single material that can replace copper. As a consequence, in copper-free brake pads the formulations have to be readjusted and reinvented. Since some of the copper functionalities are similar to the functionality of graphite, it is likely that graphite can be considered as a possible substitute for copper.

Graphite powders are commonly used in the manufacture of brake pads (resin bonded and sintered metallic) in order to provide and stabilize the required level of friction coefficient at different operating conditions, while keeping the wear rate within acceptable limits [1].

A wide range of graphite types (primary synthetic, secondary synthetic and natural) with different purity, crystallinity, particle size and shape, texture and thermal conductivity is available and used in friction materials. However, very limited literature on the influence of these properties on performance of friction materials is available. Few studies are reported on the influence of particle size of synthetic [2] and natural [3] graphite on the performance of brake pads. No investigation of the influence of graphite type on heat dissipation and on noise has been reported so far.

In this article, the effect of graphite type, texture and particle size distribution on the thermal conductivity of the brake pad and on brake squeal occurrence have been investigated. 

## 2. Experimental Section

Experimental tests are based on model friction materials with a known formulation and a reduced number of components for a better identification of the role of the graphite type. Similar to Mortelette *et al.* [4] who investigated the effect of mineral fibers on brake squeal occurrence, six simplified brake pad formulations have been prepared which contain only six ingredients (see Table 1). Apart from the reference formulation 1 without graphite, the graphite content was kept constant to 8 wt %.

**Table 1:** Brake pad test formulations.

Formulations (wt %)		1	2	3	4	5	6
Resin	Phenolic	18	18	18	18	18	18
Fibers	Mineral fibers	10	10	10	10	10	10
	Aramid pulp	5	5	5	5	5	5
Friction particles	Cashew friction dust	10	10	10	10	10	10
Filler	Barite	57	49	49	49	49	49
Graphite	KS150-600	–	8	–	–	–	–
	T150-600	–	–	8	–	–	4
	T800	–	–	–	8	–	–
	T200-2000	–	–	–	–	8	–
	C-THERM^TM^011	–	–	–	–	–	4

Three different types of graphite from TIMCAL Ltd. have been investigated: TIMREX^®^KS and TIMREX^®^T are both primary synthetic graphite, whereas TIMREX^®^C-THERM^TM^011 is a special graphite optimized for thermal conductivity in polymer compounds [5,6]. For T graphite, different particle size distributions (PSD) have been tested. C-THERM^TM^011 has been tested only in combination with T150-600 (Formulation 6) since this special graphite is meant as minor additive to boost the thermal conductivity of brake pads and not as substitute for conventional graphite that has to guarantee the lubrication and stabilization of the friction coefficient. The graphites have been characterized in terms of purity (ash content according to ASTM C561-91), particle size distribution (vibrating sieving according to ISO13320:2009), real density (measured pycnometrically by the xylene method according to DIN 51901), specific surface area (BET according to ISO9277:2010) and spring-back (Timcal internal method: Compression force is applied to a powder and the height is recorded under pressure and after pressure has been released. Spring-back is defined as the height difference in percent relative to the height under pressure).

The brake pads have been manufactured at Indian Institute of Technology according to the following manufacturing process:

(1) The various ingredients have been dry-mixed: First only aramid pulp and barite for 8 min, then mineral fibers were added and mixed for further 8 min, then phenolic resin was added and mixed for further 4 min, finally cashew friction dust and graphite were added and mixed for further 4 min;
(2) The mixtures have been hot pressed at 160 °C and 170 bars for 10 min;
(3) Afterwards all friction materials were post cured for three hours at 250 °C.

NVH study is generally complex due to interactions of the dynamics of the brake system with the excitation at the contact surface which depends on many parameters varying during braking. Since this work is organized around the microscopic nature of braking, it is important to have a test bench that can scan only those phenomena with no other interference. Noise tests have been performed on a specially designed tribometer at Laboratoire de Mécanique de Lille. The test bench consists of a rigid block on which the sample (brake pad) is positioned. This block translates on two columns via a screw nut system. The displacement of the block allows contacting the pad and the disc and to apply a defined force between the pad and disc. The dynamic behavior of the test bench is well known and reproducible. The geometry of the disc and the loading conditions are controlled. Noise measurements have been recorded at different rotational speeds of the disc and at different loadings that simulate the braking. The test procedure is described in Table 2. During linear decreasing speed tests (LDS), the velocity of the disc is linearly decreased at 50 rpm/s starting from 500 rpm and 1000 rpm and at different normal forces (250 N and 500 N). Both frequencies and intensities of noise are continuously recorded. During constant speed tests (CS), the velocity of the disc is kept constant and noise is recorded every two minutes for 20 s (9 recordings from CS1-1 to CS1-9). For each formulation, one sample was measured for a total noise test duration of about 40 min.

**Table 2:** Description of noise test procedure applied for each sample of the different brake pad formulations.

Linear decreasing speed (LDS)	LDS1-1	250 N at 500 rpm
	LDS1-2	250 N at 1000 rpm
	LDS1-3	500 N at 500 rpm
	LDS1-4	500 N at 1000 rpm
Constant speed (CS)	CS1-1 to CS1-9	250 N at 500 rpm during 15 min
Linear decreasing speed (LDS)	LDS2-1	250 N at 500 rpm
	LDS2-2	250 N at 1000 rpm
	LDS2-3	500 N at 500 rpm
	LDS2-4	500 N at 1000 rpm
Constant speed (CS)	CS2-1 to CS2-9	500 N at 1000 rpm during 20 min
Linear decreasing speed (LDS)	LDS3-1	250 N at 500 rpm
	LDS3-2	250 N at 1000 rpm
	LDS3-3	500 N at 500 rpm
	LDS3-4	500 N at 1000 rpm

From the brake pads, 10 × 10 × 3 mm^3^ samples have been cut both parallel (“through-plane”) and perpendicular (“in-plane”) to the direction of compression for thermal conductivity measurements with Laserflash equipment (Netzsch LFA447). In this method (ASTM E-1461), the front side of the sample is heated by a short light pulse. The resulting temperature rise on the rear surface is measured using an infrared detector. By analysis of the resulting temperature *vs.* time curve, the thermal diffusivity can be determined. By measuring thermal diffusivity (a) of a material, its thermal conductivity (λ) can be determined if specific heat (C_p_) and density (d) are known (λ = a·C_p_·d). Using the multiproperty measurement capabilities of LFA447 both thermal diffusivity and specific heat can be determined simultaneously on the same specimen thus yielding the thermal conductivity.

## 3. Results and Discussion

### 3.1. Graphite Characterization

The graphite properties of the lots tested in brake pad formulations are summarized in Table 3. KS and T graphite at similar particle size distribution (e.g., KS150-600 *vs.* T150-600) have overall similar properties but significant differences in terms of specific surface area. T150-600 has higher BET compared to KS150-600 due to meso and macroporosity, which indicates a larger surface roughness. A rough particle surface is beneficial for a good adhesion to the binding matrix (e.g., phenolic resin). For both KS and T, the real density is much lower than the theoretical graphite density (2.26 g/cm^3^). T and KS graphite ground to particle size <75 µm have real densities in the range 2.22–2.25 g/cm^3^, indicating that the low real densities measured in coarse graphite is mainly due to internal closed porosity. For T graphite, different particle size distributions have been tested. T200-2000 is coarser than T150-600, whereas T800 has a larger amount of fine particles (about 1/3 below 150 µm). As expected, coarser graphite has lower values of real density because of higher amount of close porosity.

C-THERM^TM^011 graphite is characterized by real density close to the theoretical graphite density and high specific surface area (BET) as an indication for high aspect ratio graphite particles. In Table 3 are also listed the spring-back values, that provide information regarding the resilience of compacted graphite powders. KS and T have similar spring-back values greater than 20%, whereas C-THERM^TM^011 has much lower values.

**Table 3:** TIMREX^®^ graphite properties (lots tested in brake pad formulations).

	KS150-600	T150-600	T800	T200-2000	C-THERM^TM^011
Ash	0.02%	0.04%	0.09%	0.06%	1.29%
Real density	2.11 g/cm^3^	2.14 g/cm^3^	2.16 g/cm^3^	2.08 g/cm^3^	2.24 g/cm^3^
BET	1.5 m^2^/g	2.7 m^2^/g	3.6 m^2^/g	2.8 m^2^/g	24.3 m^2^/g
Spring-back	28.8%	26.6%	25.2%	22.6%	12.6%
PSD (cumulative values)					
>150 µm	98.4%	96.3%	63.9%	98.3%	53.8%
>250 µm	77.2%	77.6%	50.3%	96.0%	51.2%
>500 µm	23.9%	41.1%	25.5%	82.1%	44.9%
>600 µm	0.04%	30.3%	17.0%	77.5%	41.7%
>800 µm	0.0%	0.1%	0.0%	68.3%	39.3%
>2000 µm	0.0%	0.0%	0.0%	6.9%	0.0%

### 3.2. Noise Measurements 

**Figure 1:**
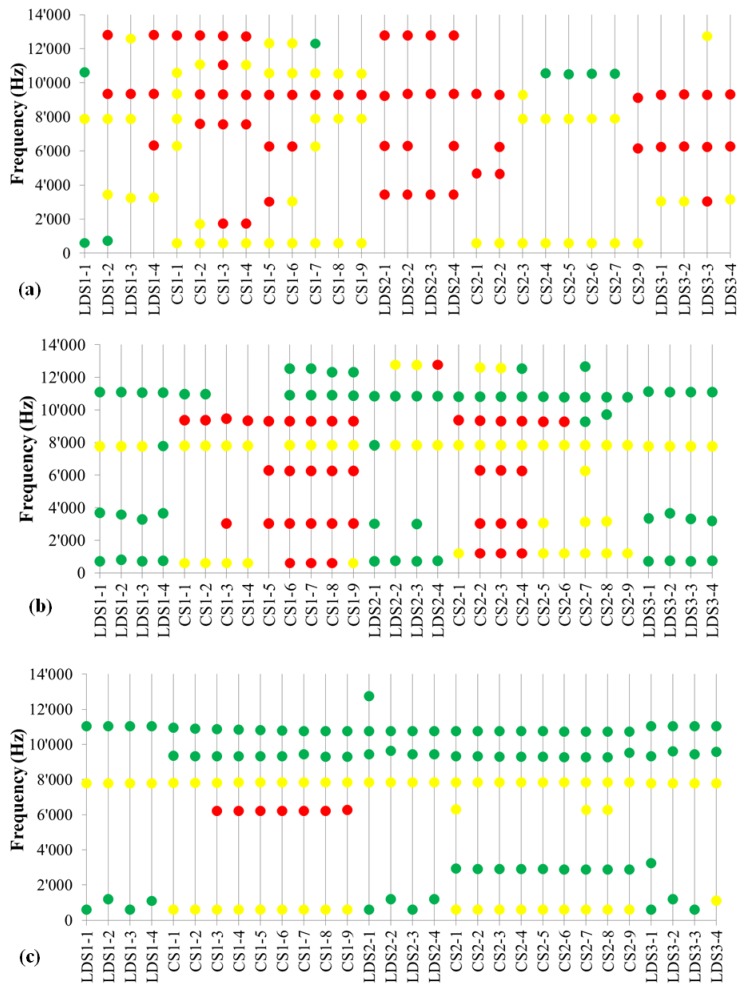
Frequencies and intensities of noise recorded during linear decreasing speed (LDS) and constant speed (CS) tests for: (**a**) Formulation 1 without graphite; (**b**) Formulation 2 with 8% KS150-600 graphite; (**c**) Formulation 3 with 8% T150-600 graphite. Green dots < 60 dB, 60 dB < yellow dots < 80 dB, red dots > 80 dB. The sequence of the test (*x*-axis) is described in Table 2.

**Figure 2:**
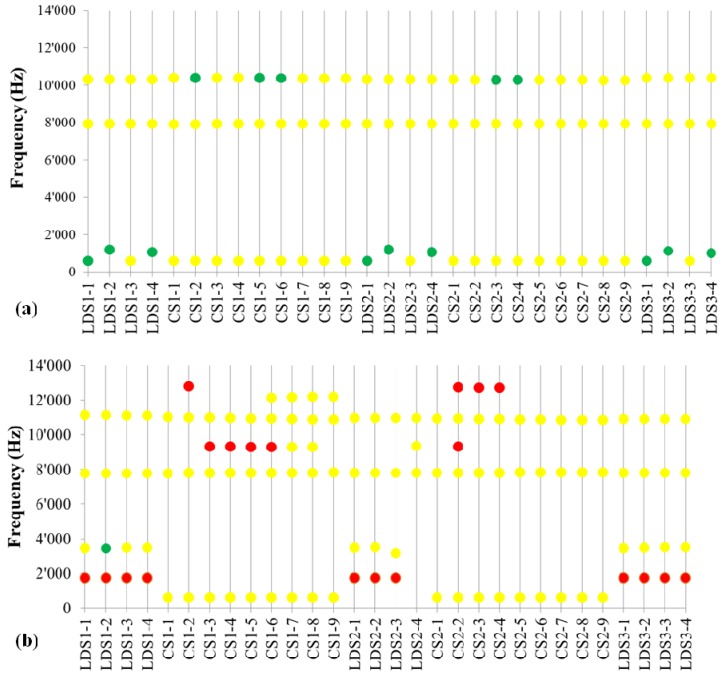

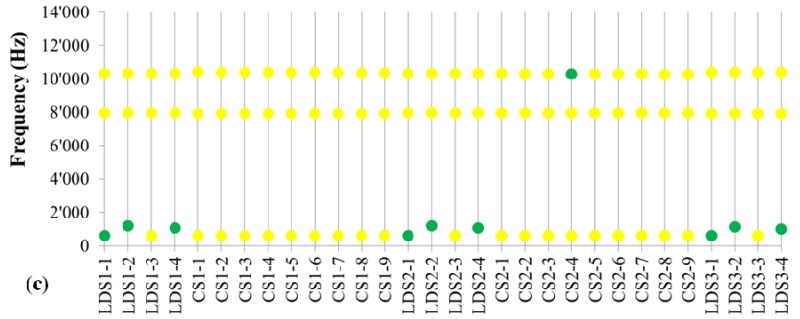
Frequencies and intensities of noise recorded during linear decreasing speed (LDS) and constant speed (CS) tests for: (**a**) Formulation 4 with 8% T800 graphite; (**b**) Formulation 5 with 8% T200-2000 graphite; (**c**) Formulation 6 with 4% T150-600 and 4% C-THERM^TM^011 graphite. Green dots < 60 dB, 60 dB < yellow dots < 80 dB, red dots > 80 dB. The sequence of the test (*x*-axis) is described in Table 2.

In Figure 1 and Figure 2 the results from noise measurements during the test described in Table 2 are summarized. Both frequencies and intensities of noise have been recorded. The intensity of the noise has been split into three categories: no noise <60 dB; low noise 60–80 dB; and high noise >80 dB.

As shown in Figure 1, the sample without graphite presents noise at various frequencies up to 13 kHz and high intensities (58 times >80 dB, only 8 times <60 dB). The substitution of part of the barite with graphite KS150-600 has a positive effect on noise: The frequencies remain about the same but the intensity is strongly reduced (39 times >80 dB, 59 times <60 dB). The use of another type of graphite T150-600 with similar particle size distribution leads to a further improvement: Only during the first constant speed test the intensity of noise is above 80 dB (at 6.2 kHz during first CS test), whereas frequencies above 8 kHz become silent (<60 dB) during test duration.

Not only the graphite type but also the particle size distribution has an influence on noise. By comparing Figure 1c to Figure 2a one observes that the noise at 6.2 kHz completely disappears with T800, that has a higher amount of fine particles (<150 µm) compared to T150-600. On the other hand, the noise at 8 kHz remains unchanged and the intensities around 10 kHz slightly increase >60 dB (but still <80dB). With the coarser graphite T200-2000 with a substantial amount of particles >800 µm the noise increases, especially at low frequency 1.7 kHz (during all LDS tests) and high frequency (9.3 kHz and 12.7 kHz during CS test), see Figure 2b. It seems that fine particles are beneficial for noise reduction. This is confirmed by Formulation 6 with 4% T150-600 and 4% C-THERM^TM^011 (fine graphite particles <150 µm), that has basically the same behavior as T800, Figure 2a and Figure 2c.

A summary of NVH test results is given in Table 4. Generally speaking, graphite contributes to noise reduction, both suppressing some noise frequencies and reducing the noise intensity. T graphite gives lower noise compared to KS graphite, which can be explained by the higher amount of surface porosity and better adhesion to the phenolic resin. Moreover, the fact that KS graphite is more isotropic compared to T graphite (as confirmed by thermal conductivity measurements—see next section) might also have an influence on the damping behavior. Our test results indicate also a beneficial effect of fine graphite particles on NVH behavior. Finally, C-THERM^TM^011 special graphite further reduces the occurrence of squeal in combination with T150-600.

**Table 4:** Summary of Noise, Vibration and Harshness (NVH) test results.

	Constant Speed	Linear decreasing speed
Formulation 1 Without Graphite	12700 Hz—92 dB 9300 Hz—106 dB 4600 Hz—90 dB 1700 Hz—96 dB	12700 Hz—95 dB 9300 Hz—106 dB 6200 Hz—91 dB3 400 Hz—86 dB
Formulation 2 8% KS150-600	9300 Hz—107 dB 6200 Hz—103 dB 3000 Hz—88 dB	12700 Hz—84 dB
Formulation 3 8% T150-600	6200 Hz—91 dB	<80 dB
Formulation 4 8% T800	<80 dB	<80 dB
Formulation 5 8% T200-2000	12700 Hz—99 dB 9300 Hz—105 dB	1750 Hz—97 dB
Formulation 6 4% T150-600 4% C-THERM^TM^011	<80 dB	<80 dB

### 3.3. Thermal Conductivity Measurements

**Figure 3:**
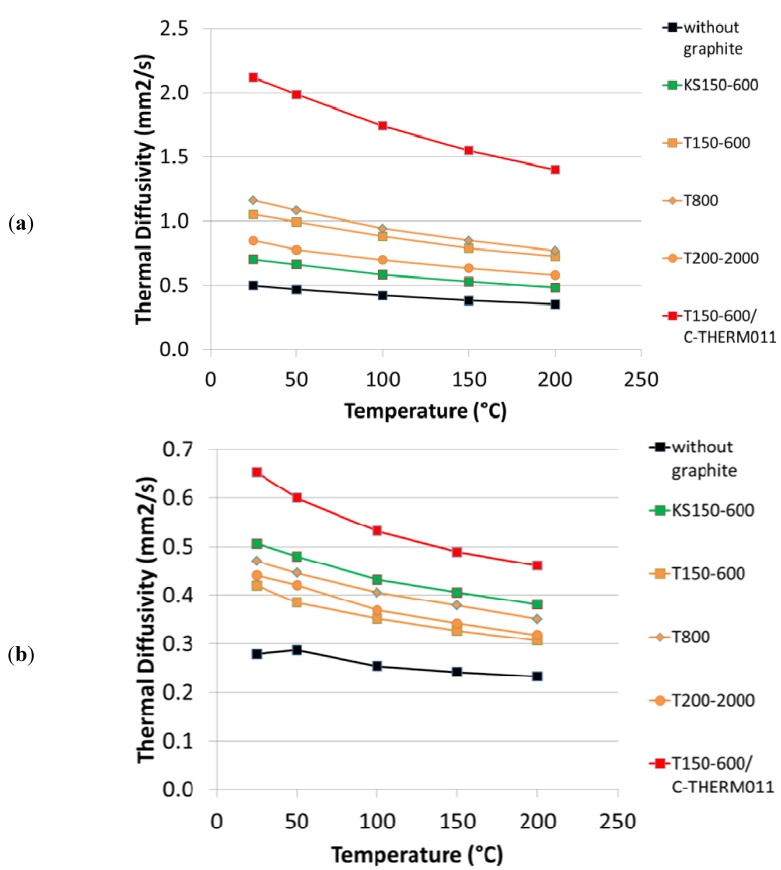
Thermal diffusivity as a function of temperature: (**a**) Measured in the direction perpendicular to the direction of compression (“in-plane”); (**b**) Measured in the direction parallel to the direction of compression (“through-plane”).

**Figure 4:**
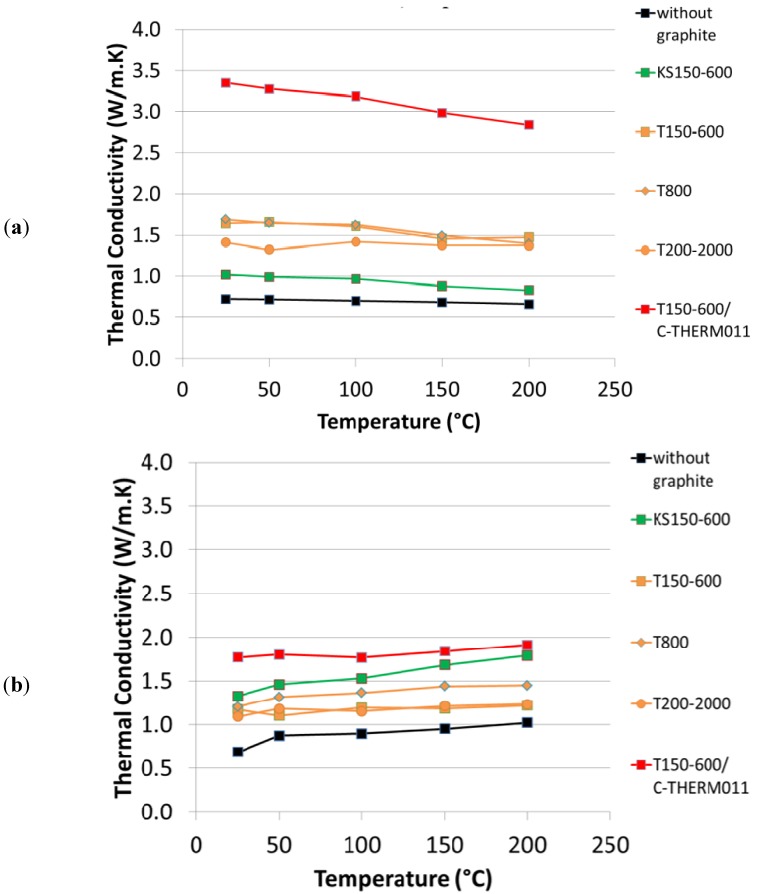
Calculated thermal conductivity as a function of temperature: (**a**) “in-plane”; (**b**) “through-plane”.

In Figure 3 the thermal diffusivity in both “in-plane” and “through-plane” directions is shown as a function of temperature for the different formulations. For all formulations one observes that the thermal diffusivity decreases with increasing temperatures. However, this effect is compensated by the increasing specific heat as a function of temperature, so that the resulting thermal conductivity is quite constant between room temperature and 200 °C (see Figure 4). For both thermal diffusivity and thermal conductivity, the “in-plane” component is larger than the “through-plane” component, due to the alignment of the graphite particles during compression molding.

**Figure 5:**
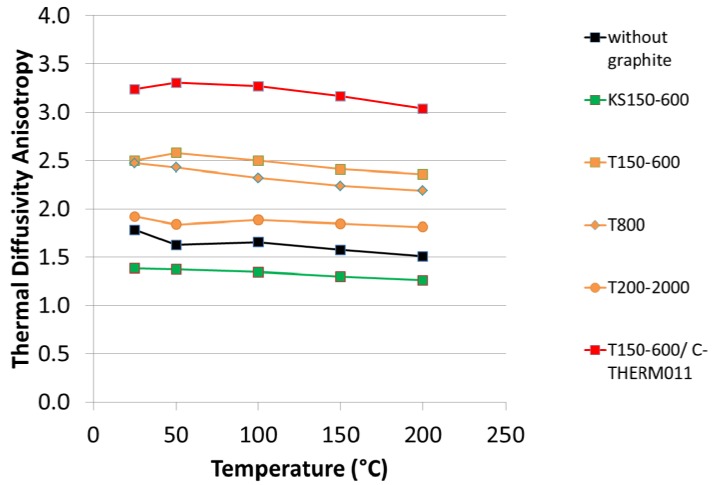
Thermal diffusivity anisotropy (“in-plane” divided by “through plane”).

All tested graphite improve the thermal conductivity compared to the formulation 1 without graphite. However, one can observe a large influence of graphite type on thermal conductivity. Synthetic graphite T150-600 gives higher in-plane but lower through-plane thermal conductivity compared to synthetic graphite KS150-600. This can be explained by the different morphology of these grades, KS being more isotropic compared to T grade. As a consequence, the thermal conductivity of brake pads containing KS graphite is much more isotropic compared to T graphite (see also Figure 5). Regarding the effect of particle size distribution, at least for T graphite there is only a small influence of PSD on thermal conductivity. However, it seems that the coarse T200-2000 leads to a lower anisotropy in thermal diffusivity.

The special graphite C-THERM^TM^011 clearly outperforms all other graphite types in terms of thermal conductivity: Compared to the formulation with 8% T150-600, the formulation with 4% T150-600 and 4% C-THERM^TM^011 has two times greater in-plane thermal conductivity. The through-plane component is also larger than in the other formulations, but the difference is not as prominent. As a consequence, the formulation with 4% T150-600 and 4% C-THERM^TM^011 has the highest thermal conductivity anisotropy, which is beneficial for an efficient heat dissipation without overheating of caliper and braking fluid.

C-THERM^TM^011 can be considered as a possible substitute of copper in order to compensate the loss of thermal conductivity in copper-free brake pads. Indeed a good thermal conductivity is essential to have a homogeneous temperature distribution in the brake pad, therefore minimizing tension cracks, hot spots and related thermo-elastic instabilities. Moreover, an efficient heat dissipation is important to stabilize the friction coefficient at high temperatures, thus reducing juddering and wear.

## 4. Conclusions

In this study, the effect of graphite on NVH performance and thermal conductivity of resin-bonded brake pads has been investigated experimentally. The following conclusions can be drawn from our test results.

Influence of graphite types on NVH behavior:

(1) Graphite has positive effect on noise reduction;
(2) Graphite with higher amount of surface roughness and better adhesion to the phenolic resin is most effective in reducing the noise (e.g., TIMREX^®^T graphite gives lower noise compared to TIMREX^®^KS). The degree of anisotropy of the graphite might also have an influence on the damping behavior;
(3) Beneficial effect of fine graphite particles on NVH behavior;
(4) C-THERM^TM^011 special graphite further reduces the occurrence of squeal in combination with T150-600.

Influence of graphite types on thermal conductivity:

(1) Graphite increases the thermal conductivity of brake pads;
(2) Minor influence of particle size distribution on thermal conductivity (for T-type synthetic graphite);
(3) Synthetic graphite T150-600 gives higher in-plane/lower through-plane thermal conductivity compared to synthetic graphite KS150-600, indicating that KS is less anisotropic compared to T;
(4) C-THERM^TM^011 clearly outperforms all other graphite types in terms of thermal conductivity;
(5) The high thermal conductivity anisotropy of brake pads containing C-THERM^TM^011 is beneficial for an efficient heat dissipation without overheating caliper and brake fluid.

In conclusion, for copper-free resin bonded brake pads, the combined use of primary synthetic TIMREX^®^T graphite with C-THERM^TM^011 graphite gives the best results in terms of NVH performance and heat dissipation. Additional tests will be performed in order to better understand the role of graphite type and particle size distribution on NVH and on the braking performance (full dynamometer tests).
